# Direct Detection of Shigella in Stool Specimens by Use of a Metagenomic Approach

**DOI:** 10.1128/JCM.01374-17

**Published:** 2018-01-24

**Authors:** Jie Liu, Mathieu Almeida, Furqan Kabir, Sadia Shakoor, Shahida Qureshi, Anita Zaidi, Shan Li, Boubou Tamboura, Samba O. Sow, Inacio Mandomando, Pedro L. Alonso, Thandavarayan Ramamurthy, Dipika Sur, Karen Kotloff, James Nataro, Myron M. Levine, O. Colin Stine, Eric Houpt

**Affiliations:** a Division of Infectious Diseases and International Health, University of Virginia, Charlottesville, Virginia, USA; b Center for Bioinformatics and Computational Biology, University of Maryland, College Park, Maryland, USA; c Aga Khan University, Karachi, Pakistan; d School of Medicine, University of Maryland, Baltimore, Maryland, USA; e Centre pour le Développement des Vaccins, Bamako, Mali; f Centro de Investigação em Saúde da Manhiça, Maputo, Mozambique; g National Institute of Cholera and Enteric Diseases, Kolkata, India; h Department of Pediatrics, University of Virginia, Charlottesville, Virginia, USA; Brigham and Women's Hospital

**Keywords:** PCR, shigella, diarrhea, metagenomics

## Abstract

The underestimation of Shigella species as a cause of childhood diarrhea disease has become increasingly apparent with quantitative PCR (qPCR)-based diagnostic methods versus culture. We sought to confirm qPCR-based detection of Shigella via a metagenomics approach. Three groups of samples were selected from diarrheal cases from the Global Enteric Multicenter Study: nine Shigella culture-positive and qPCR-positive (culture^+^ qPCR^+^) samples, nine culture-negative but qPCR-positive (culture^−^ qPCR^+^) samples, and nine culture-negative and qPCR-negative (culture^−^ qPCR^−^) samples. Fecal DNA was sequenced using paired-end Illumina HiSeq, whereby 3.26 × 10^8^ ± 5.6 × 10^7^ high-quality reads were generated for each sample. We used Kraken software to compare the read counts specific to “Shigella” among the three groups. The proportions of Shigella-specific nonhuman sequence reads between culture^+^ qPCR^+^ (0.65 ± 0.42%) and culture^−^ qPCR^+^ (0.55 ± 0.31%) samples were similar (Mann-Whitney U test, *P* = 0.627) and distinct from the culture^−^ qPCR^−^ group (0.17 ± 0.15%, *P* < 0.05). The read counts of sequences previously targeted by *Shigella/*enteroinvasive Escherichia coli (EIEC) qPCR assays, namely, *ipaH*, *virA*, *virG*, *ial*, *ShET2*, and *ipaH3*, were also similar between the culture^+^ qPCR^+^ and culture^−^ qPCR^+^ groups and distinct from the culture^−^ qPCR^−^ groups (*P* < 0.001). Kraken performed well versus other methods: its precision and recall of Shigella were excellent at the genus level but variable at the species level. In summary, metagenomic sequencing indicates that Shigella/EIEC qPCR-positive samples are similar to those of Shigella culture-positive samples in Shigella sequence composition, thus supporting qPCR as an accurate method for detecting Shigella.

## INTRODUCTION

Shigella was first recognized as the etiologic agent of bacillary dysentery or shigellosis in the 1890s and is grouped into four species: Shigella dysenteriae, S. flexneri, S. boydii, and S. sonnei. There are still more than 50,000 deaths annually from Shigella, primarily in children in developing countries (1, 2). It was identified as one of the top four pathogens causing moderate-to-severe diarrhea in the recent Global Enteric Multicenter Study (GEMS) (3). However, stool culture, as the traditional diagnostic method for Shigella, has limited sensitivity and is dependent on the quality of the specimen, its bacterial load, the type of culture media, the time of collection after the onset of diarrhea, the time of culturing after the sample collection, and utilization of antibiotics. Molecular tests, including commercially available multiplex panels, are now in wide use and have demonstrated that culture underestimates Shigella burden (4–13), for example, 2-fold in the GEMS (11). Most molecular diagnostics amplify the *ipaH* gene of Shigella, a gene that is also shared by enteroinvasive Escherichia coli (EIEC). Shigella likely constitutes the vast majority of this burden, since most specimens are positive for gene regions specific to Shigella species and enteroinvasive E. coli has not been found to be as prevalent in similar settings (4–13). We sought to utilize a non-PCR metagenomic approach to more definitively characterize Shigella-positive specimens. Our hypothesis was that culture-positive and quantitative-PCR-positive (culture^+^ qPCR^+^) samples should be similar to culture^−^ qPCR^+^ samples in terms of “Shigella” sequence.

## MATERIALS AND METHODS

### Specimens.

Twenty-seven samples were selected from diarrheal cases from three countries of the Global Enteric Multicenter Study (GEMS) (3), Mali, Mozambique, and India (nine samples each). Selection was based on budget availability and prior microbiological and molecular testing results (11, 14). All specimens from these countries that were tested by the quantitative PCR (qPCR) GEMS reanalysis were eligible for testing (11). We restricted the Shigella qPCR-positive samples to those with a cycle number of 17 to 20 in order to avoid any bias toward or against Shigella. Thereafter, nine Shigella culture^+^ qPCR^+^ samples, nine culture^−^ qPCR^+^ samples, and nine culture^−^ qPCR^−^ samples were selected randomly. The exact same aliquot of nucleic acid extract that was tested by qPCR was subjected to metagenomics sequencing. Ethics approval was obtained from the University of Maryland, all field sites, and the University of Virginia.

To evaluate the performance of metagenomic taxonomical read assignment methods, we evaluated the performance and limits of four popular tools: Kraken (15), Clark (16), MetaPhlan2 (17), and Kaiju (18). As a benchmark, we used 12 Shigella isolates selected from 11 Shigella-positive diarrheal stool specimens from a different study of childhood diarrhea conducted in Naushero Feroze, Pakistan: the Etiology, Risk Factors, and Interactions of Enteric Infections and Malnutrition and the Consequences for Child Health and Development Project (MAL-ED). Colonies were picked from xylose lysine deoxycholate agar plates inoculated directly from the swab or inoculated after overnight growth in brain heart infusion, Gram-negative, or Selenite F broth. Each isolate was identified to species by serotyping (19). One stool specimen contained isolates from two distinct species (S. flexneri and S. sonnei), and an isolate of each species was selected.

### Construction of Illumina HiSeq library.

DNA was prepared for Illumina sequencing with a KAPA high-throughput library preparation kit (Kapa Biosystems, Wilmington, MA). DNA was fragmented with the Covaris E210. Libraries were prepared using a modified version of manufacturer's with-bead protocol (Kapa Biosystems). The libraries were enriched and barcoded by 10 cycles of PCR amplification with primers containing an index sequence seven nucleotides in length.

### Metagenomic and Shigella isolate sample sequencing and processing methods.

The libraries were sequenced using paired-end Illumina HiSeq sequencing (Illumina, San Diego, CA), with a read length of 151 nucleotides and an insert size of ∼350 nucleotides. Each sample contained an average of 300 million high-quality reads prior to host removal filtering as described in Table S1 in the supplemental material. The 12 Pakistani Shigella isolates were sequenced using paired-end MiSeq Illumina sequencing with a read length of 101 nucleotides and an insert size of 350 nucleotides. In both cases, the same read quality trimming method was applied using Sickle v1.33 (https://github.com/najoshi/sickle), with a PHRED quality threshold of 30 and a minimum read length after trimming of 75 nucleotides. The sequences were deposited under BioProject PRJNA394687.

The reads from the 12 Shigella isolate libraries were assembled using Spades v3.10.0, using the “careful” mode and with “−cov-cutoff auto” to reduce the number of misassemblies. The genomes were then compared to selected representative strains of the phyletic lineages previously defined (20) and with Shigella and Escherichia NCBI reference genomes detected in high abundance by Kraken in the metagenomic samples. For that, the genomes were compared and aligned using Parsnp (21) with the “-c” parameter to constrain the use of all input genomes. The Parsnp tree generated was finally visualized using iTOL (22).

### Kraken.

Since Kraken demonstrated the best balance between precision and recall for Shigella detection, we used the Kraken software v0.10.6 with the April 2016 NCBI database version built with the “kraken-build standard” method (15). The database includes 2,787 bacteria and archaea and 4,339 viruses. We used the Kraken with the default setting with no output filtering, which provided an expected precision of 95.43% and a recall of 77.32% per the Kraken authors (15). In all cases, the reads were aligned using the “paired” mode, which uses the paired-end read information to theoretically increase the precision of Kraken by 3%.

### Metagenomic read alignment to Shigella/EIEC-specific virulence genes.

To improve the precision of Shigella detection, we further examined the counts of six Shigella/EIEC-specific virulence genes, including *ipaH*, *ShET2*, *ial*, *virA*, *virG*, and *ipaH3*, as well as the markers in the Mxi-Spa-ipa region (23), in these metagenomic samples. To avoid any impact of fragment length, a fragment that was exactly 1,000 nucleotides was analyzed for each gene, except for *ipaH3* (where 531 nucleotides was analyzed to maintain uniqueness [see Table S2 in the supplemental material]). The metagenomic reads were then aligned to these 1-kb virulence genes using bowtie2 using the paired-end end-to-end read mode assignment; i.e., a read is assigned to an amplicon only if both pairs align entirely on the same amplicon.

### Statistics.

We used Mann-Whitney U test to examine whether the read counts of Shigella species, human sequences, or virulence factors in the culture^−^ qPCR^+^ samples were different from the culture^+^ qPCR^+^ or culture^−^ qPCR^−^ samples. Correlation of read counts between various virulence factors was tested by regression analysis using analysis of variance (ANOVA). Two-tailed *P* values were calculated, and values of <0.05 were considered statistically significant. All analyses were performed using IBM SPSS version 24.

### Accession number(s).

Sequences were deposited under BioProject PRJNA394687, accession numbers SRX3008896 to SRX3008922.

## RESULTS

### Direct detection of Shigella in stool samples by metagenomics sequencing.

The 27 selected diarrheal samples were previously tested with a broad range of pathogen-specific qPCRs utilizing TaqMan Array Cards as described previously (11). Shigella/EIEC was the primary diarrhea-associated pathogen (i.e., with the highest odds ratio for diarrhea) in both the culture^+^ qPCR^+^ and the culture^−^ qPCR^+^ groups (average quantification cycles of 18.7 ± 1.03 versus 18.7 ± 1.05; *P* = 0.594). Additional diarrhea-associated pathogens were identified in specimens, as indicated in Table S3 in the supplemental material, and included rotavirus, Cryptosporidium spp., astrovirus, Helicobacter pylori, and adenovirus 40/41.

**FIG 1:**
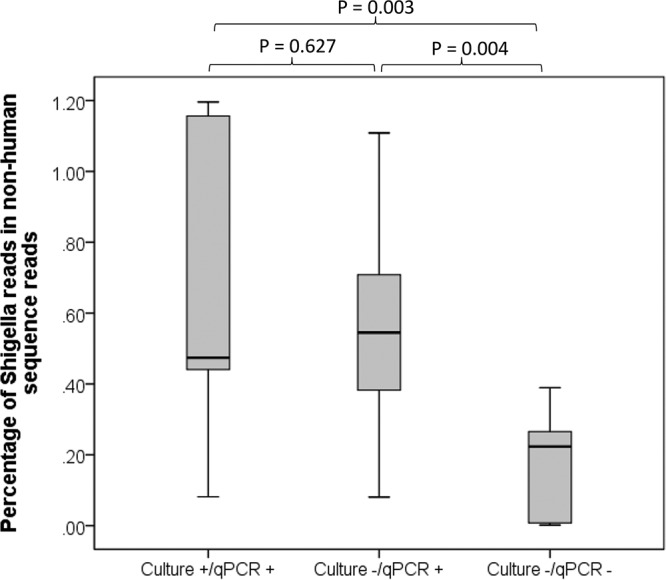
Proportion of read counts assigned to Shigella species by Kraken. The percentage was calculated by dividing the Shigella reads with the nonhuman sequence reads in a given sample.

The 27 metagenomic libraries had an average of 300M high-quality paired-end reads. There was no difference in total reads among the three sample groups (295.4 ± 42.8 × 10^**6**^ for Shigella culture^+^ qPCR^+^ samples, 295.4 ± 47.7 × 10^**6**^ for Shigella culture^−^ qPCR^+^ samples, and 329.5 ± 59.2 × 10^**6**^ for Shigella culture^−^ qPCR^−^ samples; *P* > 0.05). However, nearly half of the reads (41.8 ± 39.5%) were human sequences and varied from 0.3 to 97.8% of the reads in individual libraries. Shigella culture^−^ qPCR^−^ samples had a much lower composition of human sequences (13.2 ± 20.0%, *P* < 0.05) than the other two groups of samples (61.7 ± 43.1% for Shigella culture^+^ qPCR^+^ samples and 50.3 ± 37.0% for Shigella culture^−^ qPCR^+^ samples; *P* = not significant). Shigella qPCR^+^ samples had high read counts of human sequences (82.4% for the 4 dysenteric samples and 58.5% for the 14 nondysenteric samples; *P* = 0.142). To adjust for potential sampling bias introduced by the variable human sequence composition, the read counts were compared as the proportion of nonhuman sequence reads (Fig. 1). Overall, 0.65 ± 0.42% of nonhuman sequence reads were assigned by Kraken as Shigella species for culture^+^ qPCR^+^ samples and 0.55 ± 0.31% for culture^−^ qPCR^+^ samples (*P* > 0.05). Both were significantly higher than those of culture^−^ qPCR^−^ samples (0.17 ± 0.15%, *P* < 0.05).

**FIG 2:**
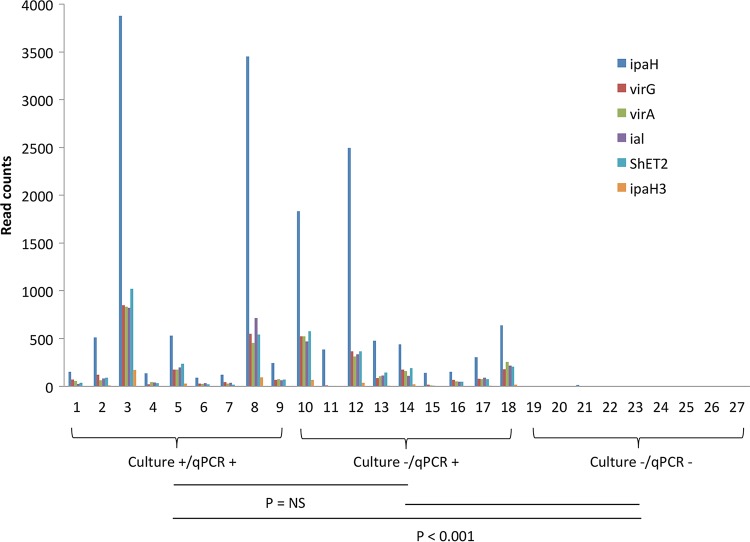
Read counts of six Shigella virulence genes in 27 stool samples. A 1-kb region was interrogated for each target, except for 531 bp for *ipaH3*. The sample order remained the same as in Table S1 in the supplemental material. NS, not significant.

The read counts of Shigella/EIEC-specific virulence genes *ipaH*, *ipaH3*, *ial*, *ShET2*, *virA*, and *virG* are shown in Fig. 2. For all six gene targets, culture^−^ qPCR^+^ samples yielded read counts similar to those of the Shigella culture^+^ qPCR^+^ samples, whereas few reads were generated among Shigella culture^−^ qPCR^−^ samples. A high correlation was observed between these genes (e.g., *R*^2^ = 0.889 to 0.932 between *ipaH* and any of the other 5 genes). The read counts of 18 *ipaH*-positive samples showed low correlation (e.g., *R*^2^ = 0.0288, 0.006 for *ipaH* and *virA*, respectively) with the corresponding *ipaH* qPCR quantification cycles; however, this improved when normalized to nonhuman sequence reads (e.g., *R*^2^ = 0.259 and 0.361). Unfortunately, the genes responsible for biosynthesis of Shigella O antigen are common to many other bacteria and thus cannot be used to detect or distinguish Shigella from EIEC (24). There is, however, another region, Mxi-Spa-ipa, that has been reported to have some ability to discriminate Shigella from EIEC (23), and these genes showed a similar pattern of positivity among *ipaH* PCR^+^ specimens and negativity among *ipaH* PCR^−^ specimens, supporting the idea that these *ipaH* PCR^+^ specimens were from Shigella (see Table S4 in the supplemental material).

### Testing Kraken with whole-genome sequences of Shigella isolates.

**FIG 3:**
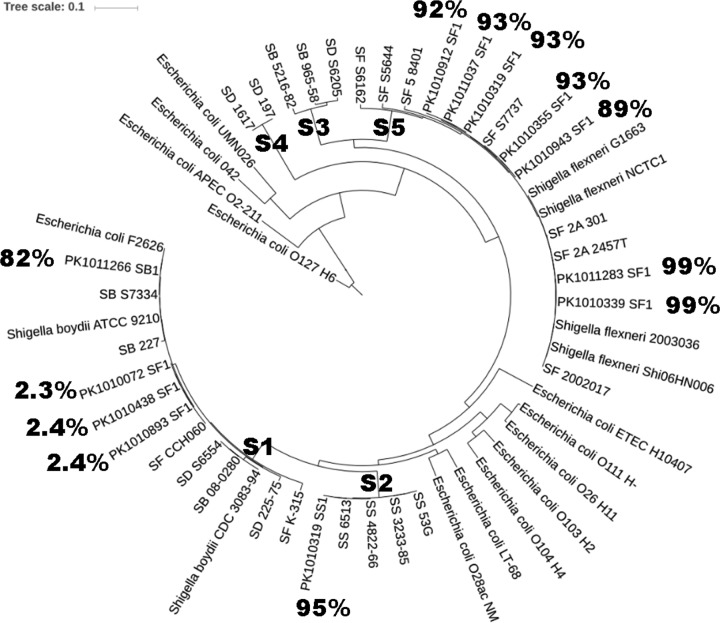
Phylogenetic tree of selected isolates of Shigella spp. The Shigella lineages were determined previously (20). The Pakistani isolates are denoted by PK*. SF, S. flexneri; SD, S. dysenteriae; SS, S. sonnei; SB, S. boydii. The percentage displays the Kraken annotation precision at the species level for Shigella.

In order to validate the precision and recall of Kraken for Shigella at the genus and species level, we tested the 12 DNA sequences from well-characterized isolates of Shigella from Pakistan. At the Shigella genus level, the Kraken precision ranged from 86 to 99.2% (corresponding to the proportion of reads assigned to Shigella out of the reads classified to a genus [15]). At the species level, the precision was either close to 2% or above 80% (Table 1). As shown in Fig. 3, all of the low precision occurred among S. flexneri isolates that were in the S1 phyletic lineage (the S1 lineage includes S. flexneri, S. dysenteriae, and S. boydii as described previously [20]). In contrast, species precision for S. sonnei and S. flexneri of the S2 and S5 lineages, respectively (that contain one nominal species), was >90%. Accordingly, among the nine culture^+^ qPCR^+^ stool samples, four S. sonnei (S2 lineage) and three S. flexneri (all of S5 lineage) culture-positive specimens were correctly identified by metagenomics sequencing as those with the highest read count, whereas two S. flexneri samples (1 of S1 lineage) were misclassified as other Shigella species (see Table S5 in the supplemental material).

**TABLE 1:** Benchmark of Kraken's precision and sensitivity for isolates of Shigella spp.

Isolate	Species identified by culture	Phyletic lineage^*a*^	No. of reads (Mb)	Estimated coverage	Precision or sensitivity (%)^*b*^			
					Genus precision	Genus sensitivity	Species precision	Species sensitivity
PK1010072	S. flexneri	S1	4.3	95.8	86.8	38.4	2.3	0.8
PK1010893	S. flexneri	S1	9.6	214.1	86.7	37.4	2.4	0.9
PK1011266	S. boydii	S1	10.4	231.5	87.4	39.3	2.4	0.9
PK1010319	S. flexneri	S1	16.4	364.8	88.7	40.0	82.7	30.4
PK1010319	S. sonnei	S2	6.5	144.1	96.9	36.0	95.1	28.3
PK1010339	S. flexneri	S5	5.1	112.6	96.2	50.6	93.4	40.5
PK1010355	S. flexneri	S5	7.0	154.6	99.2	54.1	98.7	43.8
PK1010912	S. flexneri	S5	10.1	224.3	94.0	46.7	92.9	38.9
PK1010943	S. flexneri	S5	3.1	69.0	95.1	47.6	92.2	40.0
PK1010438	S. flexneri	S5	14.4	320.8	91.5	45.9	88.7	38.4
PK1011037	S. flexneri	S5	9.7	215.7	95.8	47.9	93.4	40.4
PK1011283	S. flexneri	S5	10.4	230.0	99.1	53.5	98.6	43.6

a Phyletic lineage was assigned according to the system of Sahl et al. (20).

b Sensitivity refers to the proportion of sequences assigned to the correct Shigella genus or species. Precision, i.e., the positive predictive value, refers to the proportion of correct classifications, out of the total number of classifications attempted.

We further compared Kraken to other tools, including Clark, MetaPhlan2, and Kaiju (see Table S6 in the supplemental material). Kraken and Clark (93.2 ± 4.7% versus 91.7 ± 5.6%; Wilcoxon signed-rank test, *P* < 0.05) outperformed MetaPhlan2 (66.7 ± 33.1%, *P* < 0.05) and Kaiju (67.5 ± 9.6%, *P* < 0.05) for precision. For sensitivity, although MetaPhlan2 showed good performance for the S2 and S5 lineages (74.2 ± 13.4%), its recall rate was poor for the S1 lineage (2.1 ± 0.3%). In contrast, Kraken showed consistent recall rates across lineages (47.8 ± 5.6% for S2 and S5 and 38.8 ± 1.2 for S1), higher than both Clark (28.3 ± 4.9%, *P* < 0.05) and Kaiju (10.7 ± 2.3%, *P* < 0.05).

### Detection of other pathogens through metagenomics sequencing.

In addition, we examined whether the detection of DNA viruses and certain other bacterial pathogens by qPCR (see Table S3 in the supplemental material) could also be supported by the metagenomic results. For many pathogens, such as Campylobacter jejuni and C. coli, adenovirus 40/41, enteroaggregative E. coli (EAEC), and typical enteropathogenic E. coli (EPEC), the pathogen-specific read counts identified by Kraken demonstrated a quantitative relationship with qPCR quantification cycle (*C_q_*) values of the relevant target genes (see Fig. S1 in the supplemental material). However, there was also substantial metagenomic background in qPCR-negative specimens.

## DISCUSSION

The main finding of this work is that the metagenomic composition of Shigella qPCR-positive samples is similar to that of culture-positive samples. In other words, the Shigella qPCR results based on *ipaH* appear to be robust even if cultures are negative. Our result is consistent with those of Lindsay et al. (10) that showed that the culture^−^ qPCR^+^ samples were indistinguishable from culture^+^ qPCR^+^ samples by clinical criteria.

In the metagenomic analysis, we examined the read counts of known Shigella/EIEC-specific virulence genes. We surveyed *ipaH*, *ial*, *virA*, *virG*, *ShET2*, and *ipaH3. ipaH* is the most commonly used target gene for diagnostic PCR assays and is present in multiple copies on both chromosome and plasmid (13). *ial*, *virA*, *virG*, and *ShET2* are found in single copy on the plasmid (25–27), whereas *ipaH3*, which is a conserved region previously identified to distinguish Shigella from the majority of E. coli, is present in single copy on the chromosome (20). The metagenomic read counts of these genes were highly correlated with each other within a sample, which is consistent with our previous results (9, 11). The read counts distinguished the Shigella qPCR positives from negative and were similar between culture^+^ qPCR^+^ and culture^−^ qPCR^+^ samples. As expected, *ipaH* had the highest read counts for all the Shigella qPCR-positive samples, usually 5- to 7-fold higher than *ial*, *virA*, *virG*, and *ShET2*. In contrast, *ipaH3* had the lowest read counts, usually 20- to 30-fold lower than that of *ipaH*. This confirmed that *ipaH* is likely the most efficient available target for PCR detection of Shigella species.

As a benchmark comparison, we tested 12 genome sequences from Shigella isolates carefully identified to the species level to determine the precision and recall for Kraken, Clark, MetaPhlan2, and Kaiju. Kraken outperformed for both precision and recall at the genus level. We also tested the assignment of reads to the species level. The Shigella genus includes four nominal species—S. sonnei, S. flexneri, S. dysenteriae, and S. boydii—that can be accurately distinguished by serotyping. The precision for the genus was very good (**≥**86%) and, although the precision for the species could also be very good (**≥**88%), sometimes it was extremely poor (∼2.4%). The precision of the sequences was dependent upon the position of the sequence in the phylogenetic tree (Fig. 3). The previously defined phyletic lineage S1 (20) was the source of all of the assignments of sequences that had low precision. This lineage, S1, is characterized by the presence of three nominal species: S. flexneri, S. dysenteriae, and S. boydii. Thus, in this case (and in the S3 lineage, where S. dysenteriae and S. boydii co-occur) (20, 28), all programs, including Kraken, that assume a monophyletic lineage for its assignment (29) produce low precision. However, for species such as S. sonnei that are monophyletic, the assignment to species is very good.

Another observation was that Shigella qPCR-positive samples yielded a much higher proportion of human sequence reads. We suspect this is because clinical shigellosis is invasive and characterized by the presence of blood, mucus, and epithelial disruption. Shigella was most likely the causative agent in these samples because of its strong association with diarrhea at high quantity, even if in the presence of other pathogens (7, 10, 11).

This study had limitations, such as the small sample size (limited by cost), the depth of metagenomic sequencing, the challenges posed by variable levels (up to 97.9%) of human DNA in fecal samples, low Shigella abundance (0.27 ± 0.32%), the high level of sequence similarity of Shigella genome with E. coli genome in available databases, and imperfect S. flexneri species assignment by Kraken. Nonetheless, our study clearly demonstrates Shigella *ipaH* qPCR-positive specimens contain genes from Shigella, whereas qPCR-negative specimens do not. Thus, culture misses true cases of Shigella and molecular diagnosis with appropriate quantitative cutoffs provides a more accurate method for detecting Shigella.

## Supplementary Material

Supplemental material
