# Supplementary material for: Direct Detection of Shigella in Stool Specimens by Use of a Metagenomic Approach
Source: J Clin Microbiol. 2018 Jan 24;56(2):e01374-17. doi: 10.1128/JCM.01374-17 (PMC5786726; doi:10.1128/JCM.01374-17)
Supplement: Supplemental material [file supp_56_2_e01374-17__index.html]

Supplemental material 

# Direct Detection of Shigella in Stool Specimens by Use of a Metagenomic Approach

## Supplemental material

- Supplemental file 1 -

  Tables S1 (Read counts of 27 stool samples subjected to metagenomics sequencing), S2 (Interrogated region of 6 *Shigella* virulence genes), S3 (Pathogen detection in 27 selected diarrheal samples, shown as Cq values), S4 (Metagenomic sequencing reads of markers in the Mxi-Spa-Ipa region), S5 (Raw read counts of *Shigella* species in culture-positive/qPCR-negative samples), and S6 (Comparison of precision and recall at the genus level between Kraken and Clark, MetaPhlan2, and Kaiju for *Shigella* isolates) and Fig. S1 (Comparison of metagenomics sequencing reads and qPCR Cq values)

  PDF, 474K
