# Supplementary material for: Direct Detection of Shigella in Stool Specimens by Use of a Metagenomic Approach
Source: J Clin Microbiol. 2018 Jan 24;56(2):e01374-17. doi: 10.1128/JCM.01374-17 (PMC5786726; doi:10.1128/JCM.01374-17)
Supplement: Supplemental material [file JCM.01374-17_zjm999095800s1.pdf]

Supplemental Table S1. The read counts of the 27 stool samples subjected to metagenomics sequencing. MAL = Mali, MOZ = Mozambique, IND = India.

Sample	Sample ID	Site	Culture	qPCR	Total high quality reads
1	202260	MAL	+	+	255853444
2	203032	MAL	+	+	370748896
3	203745	MAL	+	+	336959130
4	300244	MOZ	+	+	267173944
5	302912	MOZ	+	+	318564818
6	303061	MOZ	+	+	275670712
7	500242	IND	+	+	251507850
8	504039	IND	+	+	322336854
9	503617	IND	+	+	259519220
10	202455	MAL	-	+	273141504
11	202742	MAL	-	+	304390100
12	203725	MAL	-	+	389080142
13	300055	MOZ	-	+	293291234
14	300716	MOZ	-	+	254650088
15	302016	MOZ	-	+	347812514
16	500620	IND	-	+	282549862
17	503417	IND	-	+	231606090
18	504637	IND	-	+	282331220
19	200714	MAL	-	-	373848930
20	201361	MAL	-	-	357411820
21	202587	MAL	-	-	271984392
22	300056	MOZ	-	-	387004856
23	300763	MOZ	-	-	244645304
24	302001	MOZ	-	-	409672600
25	503034	IND	-	-	327307332
26	503626	IND	-	-	258594624
27	504241	IND	-	-	335360728

Supplemental Table S2. The interrogated region of 6 *Shigella* virulence genes.

**Gene**	**Function**	**Accession number**	**Location**
ipaH	invasion plasmid antigen H	CP000039	186587 - 185588
ipaH3	invasion plasmid antigen H3	CP000038	1391081 - 1391611
ial	invasion associated protein	D50601	17450 - 18449
ShET2	enterotoxin Set2	Z54211	491 - 1490
virA	microtubule disruption	D26468	1101 - 2100
virG	encode icsA involved in actin based mobility and intercellular spread	M22802	2060 - 3059

Supplemental Table S3. Pathogen detection in 27 selected diarrheal samples, shown as quantification cycle (Cq). Red represents highly diarrhea-associated quantities, orange diarrhea-associated quantities, yellow represents detection with no association or for pathogens where no Cq cutoff exists (Liu, 2016), and grey denotes no detection.

	Diarrhea associated pathogen with a Cq cutoff													Pathogen without a Cq cutoff or diarrea association									
Sample	Shigella/EIEC	adenovirus 40/41	C. jejuni/coli	H. pylori	Salmonella	V. cholerae	tEPEC	ST-ETEC	astrovirus	norovirus GII	rotavirus	sapovirus	Cryptosporidium	norovirus GI	Aeromonas	B. fragilis	C. difficile	EAEC	aEPEC	LT-ETEC	Ascaris	E. bieneusi	Giardia
Shigella culture + / qPCR +	19	-	23	-	-	-	21	-	28	-	-	-	-	-	-	-	-	18	-	-	-	30	20
	19	-	34	-	-	-	34	-	-	-	28	-	-	-	-	-	-	-	-	34	-	-	-
	20	-	-	-	-	-	-	25	-	29	-	-	29	-	-	-	-	21	-	-	-	-	31
	18	34	-	-	-	-	-	-	-	-	-	-	-	-	-	-	-	-	33	-	-	-	34
	17	15	-	35	-	-	27	24	-	-	-	33	32	-	-	27	-	29	-	-	-	23	18
	20	26	-	-	-	-	-	31	-	-	-	-	-	-	32	-	-	22	31	-	-	-	32
	19	26	-	-	-	-	-	-	-	-	-	-	-	-	-	31	-	32	-	-	-	-	30
	17	35	-	-	-	-	-	-	19	-	35	-	-	21	-	-	-	-	-	-	-	-	33
	19	28	-	-	-	-	-	-	-	-	-	-	-	-	-	-	-	-	-	-	-	-	31
Shigella culture - / qPCR +	18	-	-	-	-	-	18	-	-	-	-	-	-	-	-	-	-	-	-	-	-	-	32
	19	-	-	-	-	-	13	35	-	31	-	-	-	-	-	33	-	28	-	-	-	-	35
	20	-	-	-	-	-	29	-	-	-	-	-	-	-	-	-	-	23	-	-	-	-	31
	19	-	-	-	-	-	34	-	-	-	-	-	-	-	-	-	-	16	-	34	-	-	32
	17	32	-	-	-	35	34	-	-	-	-	-	-	-	-	-	-	29	-	-	-	-	15
	20	-	-	-	-	-	34	-	-	-	-	-	-	-	-	-	-	18	-	-	-	-	-
	18	-	-	-	-	-	-	-	-	-	-	-	-	-	-	-	-	-	31	-	-	-	21
	19	-	-	34	-	-	-	-	-	-	-	-	-	-	-	21	-	18	-	-	-	-	-
	17	-	25	-	-	-	-	-	-	-	-	-	32	-	-	27	-	-	26	27	29	-	19
Shigella culture - / qPCR -	-	-	-	-	-	-	-	-	-	-	-	29	30	-	-	-	34	-	-	-	-	-	22
	-	29	26	-	-	-	32	-	-	33	29	-	-	-	-	-	-	-	-	28	-	33	15
	-	-	-	-	34	-	-	-	-	-	24	-	17	35	-	-	-	35	-	-	-	-	20
	-	33	-	-	-	-	-	32	-	-	-	29	-	-	-	-	-	19	-	-	-	35	32
	-	-	-	-	-	-	-	-	-	-	24	31	-	-	-	-	-	34	30	-	-	35	24
	-	31	-	-	-	-	35	32	-	-	-	-	24	-	-	-	-	16	-	-	-	-	24
	-	-	-	-	-	-	-	-	21	-	-	-	-	-	-	-	-	22	23	-	-	-	34
	-	-	19	28	-	-	-	17	-	26	-	-	-	-	32	-	-	-	22	-	-	-	32
	-	17	28	-	-	-	19	-	-	-	-	31	-	-	-	-	-	19	-	-	-	-	25

Supplemental Table S4 Metagenomic sequencing reads of the markers in the Mxi-Spa-Ipa region (Hazen, 2016). Specimens 1-9 are culture+/qPCR+, 10-18 are culture-/qPCR+, 19-27 are culture-/qPCR-.

sampleID	ipaJ	virB	acp	ipaA	ipaD	ipaC|Mxi-Spa	ipaB|Mxi-Spa	ipgC	ipgB1|Mxi-Spa	ipgA	icsB	ipgD	ipgE	ipgF	mxiG	mxiH|Mxi-Spa	mxiI|Mxi-Spa	mxiJ|Mxi-Spa	mxiK|Mxi-Spa	mxiN|Mxi-Spa	mxiL|Mxi-Spa	mxiM|Mxi-Spa	mxiE	mxiD	mxiC|Mxi-Spa	mxiA|Mxi-Spa	spa15|Mxi-Spa	spa47	spa13|Mxi-Spa	spa32|Mxi-Spa	spa33|Mxi-Spa	spaP	spa9|Mxi-Spa	spa29|Mxi-Spa	spa40
1	28	44	2	116	30	52	104	8	24	4	74	110	4	6	66	2	0	32	12	16	6	14	30	110	62	130	4	64	0	42	22	28	2	20	76
2	52	74	2	150	60	98	174	16	38	2	112	108	16	12	62	2	0	50	24	34	10	20	38	146	64	142	12	132	0	52	56	34	2	50	86
3	766	662	22	1862	994	1266	1936	266	380	128	1390	1640	126	234	784	46	124	584	354	412	172	208	432	1522	880	1990	232	1514	122	590	492	484	70	500	638
4	16	28	0	68	30	46	68	10	6	2	30	76	0	10	42	0	0	10	6	26	4	2	4	58	32	88	10	30	2	16	18	10	0	24	36
5	160	190	8	528	220	228	478	84	54	32	334	384	16	42	232	2	10	118	74	116	36	40	76	454	230	470	32	284	16	186	166	118	0	146	174
6	14	18	0	52	40	24	46	6	10	4	40	44	0	0	18	2	0	8	4	10	2	2	22	42	12	52	0	26	2	12	8	16	0	16	28
7	22	14	0	56	26	40	46	6	10	0	42	44	0	2	12	0	0	12	4	12	6	4	14	54	28	42	2	48	8	14	14	18	2	12	24
8	480	622	28	1620	682	692	1342	204	290	112	1222	1340	96	158	814	30	30	444	214	484	134	196	306	1484	888	1814	122	962	74	530	436	408	26	454	596
9	48	52	0	162	68	84	152	8	12	14	124	144	12	14	70	0	4	52	16	24	4	20	6	156	100	150	4	96	0	46	32	26	0	34	54
10	514	448	4	1224	646	684	1244	114	250	72	838	980	60	102	654	8	36	328	158	286	94	126	220	1054	640	1320	96	770	26	414	324	296	8	300	520
11	4	6	0	6	0	2	0	0	0	2	16	2	0	0	2	0	2	2	2	2	0	0	0	0	2	4	0	8	0	4	0	0	0	2	8
12	206	210	0	610	306	436	644	88	134	26	366	550	42	60	260	2	26	180	102	126	44	24	80	482	258	652	32	528	14	168	126	118	8	160	234
13	50	98	2	268	122	96	226	22	58	10	180	198	16	36	110	6	16	70	24	54	18	14	56	228	130	218	12	156	10	82	58	74	4	50	110
14	98	100	0	308	176	98	284	22	52	8	220	232	8	26	70	0	6	66	18	44	12	20	44	196	148	266	8	218	2	68	40	90	0	78	86
15	6	2	0	10	4	10	22	0	10	0	2	10	0	0	10	0	0	0	4	4	0	2	0	16	4	16	0	8	0	2	0	4	0	0	0
16	40	62	0	96	50	64	122	10	30	6	92	118	0	6	70	0	4	38	2	32	6	10	18	100	58	142	8	50	4	44	42	36	0	28	58
17	62	100	0	230	50	104	196	20	30	6	148	206	14	28	82	0	6	40	42	84	18	12	42	184	94	186	16	148	4	86	58	50	0	64	114
18	156	170	2	438	186	222	334	60	108	48	306	304	30	48	198	0	6	118	64	84	48	30	78	340	196	480	40	266	26	146	96	98	0	122	210
19	4	2	0	0	2	2	0	0	0	0	2	0	0	2	2	0	0	0	0	0	0	2	0	2	2	8	0	0	0	0	2	0	0	0	0
20	2	0	0	0	4	0	0	0	0	0	2	0	0	0	0	0	0	2	0	2	0	0	0	4	0	0	0	2	0	0	0	0	0	2	0
21	0	4	0	12	8	0	2	0	2	0	8	4	0	4	2	0	0	2	0	2	0	2	0	2	8	6	0	10	0	8	0	2	0	4	0
22	0	0	0	0	0	0	0	0	0	0	0	0	0	0	0	0	0	0	0	0	0	0	0	0	0	0	0	0	0	0	0	0	0	0	0
23	0	0	0	0	0	0	0	0	0	0	0	0	0	0	0	0	0	0	0	0	0	0	0	0	0	0	0	0	0	0	0	0	0	0	0
24	0	0	0	0	0	0	0	0	0	0	0	0	0	0	0	0	0	0	0	0	0	0	0	2	0	0	0	0	0	0	0	0	0	0	0
25	0	0	0	0	0	0	0	0	0	0	0	0	0	0	0	0	0	0	0	0	0	0	0	0	0	0	0	0	0	0	0	0	0	0	0
26	0	0	0	0	0	0	0	0	0	0	0	0	0	0	0	0	0	0	0	0	0	0	0	0	0	0	0	0	0	0	0	0	0	0	0
27	0	0	0	0	0	0	0	0	0	0	0	0	0	0	0	0	0	0	0	0	0	0	0	0	0	0	0	0	0	0	0	0	0	0	0

Supplemental Table S5. The raw read counts of *Shigella* species in culture+/qPCR+ samples. Highest read counts are bolded if correctly classified the stool culture result and underlined if misclassified the stool culture result.

**Culture identification**	***S. flexneri***	***S. boydii***	***S. sonnei***	***S. dysenteriae***
S. flexneri serotype 6	8606	41114	10176	5659
S. flexneri serotype 3a	1083131	455181	739325	1124807
S. sonnei	32255	58086	**929797**	25220
S. flexneri serotype 2a	**32371**	668	2009	1528
S. sonnei	6125	6917	**164642**	2292
S. flexneri serotype 2a	**51050**	10431	14378	23872
S. flexneri serotype 3a	**21916**	661	2422	400
S. sonnei	8523	30985	**706606**	7810
S. sonnei	2738	3079	**62117**	591

Supplemental Table S6. Comparison of precision and recall on the genus level between Kraken and Clark MetaPhlan2, Kaiju on the *Shigella* isolates. The highest precision or sensitivity for each isolate was bolded.

	Monophyletic	Genus precision				Genus sensitivity			
Isolate ID	lineage	Kraken	Clark	MetaPhlan2	Kaiju	Kraken	Clark	MetaPhlan2*	Kaiju
PK1010072_SF	S1	**86.8**	84.2	5.9	54.9	**38.4**	23.5	1.7	8.6
PK1010438_SF	S1	**86.7**	83.8	35.8	55.6	**37.4**	22.7	2.3	8.4
PK1010893_SF	S1	**87.3**	85.2	29.8	56.6	**39.3**	24.6	2.1	8.9
PK1011266_SB	S1	**88.7**	87.4	29.2	60.1	**40.0**	24.8	2.2	9.2
PK1010319_SS	S2	96.9	**97.1**	55.7	63.5	36.0	21.9	**47.6**	7.4
PK1010319_SF	S5	**96.2**	94.5	94.4	75.4	50.6	33.0	**90.6**	12.7
PK1010339_SF	S5	99.2	**99.4**	93.5	81.9	54.1	35.9	**81.8**	14.2
PK1010355_SF	S5	**94.9**	94.0	89.1	73.3	46.7	29.2	**68.6**	11.2
PK1010912_SF	S5	**95.1**	92.7	90.6	70.5	47.6	29.8	**81.6**	11.4
PK1010943_SF	S5	**91.5**	89.4	92.5	66.0	45.9	28.6	**65.0**	10.9
PK1011037_SF	S5	**95.8**	93.6	90.7	71.4	47.9	30.3	**80.6**	11.6
PK1011283_SF	S5	99.1	**99.4**	93.6	81.1	53.5	35.6	**77.6**	14.2

*metaphlan2 gives an estimation of the number of reads that should match that taxa based on the marker gene detection

Supplemental Figure S1. Comparison of metagenomics sequencing reads versus qPCR Cq values. Only DNA pathogens with more than 5 positives were plotted, i.e. qPCR positive with Cq < 35. Regression was fit for Ct < 30.

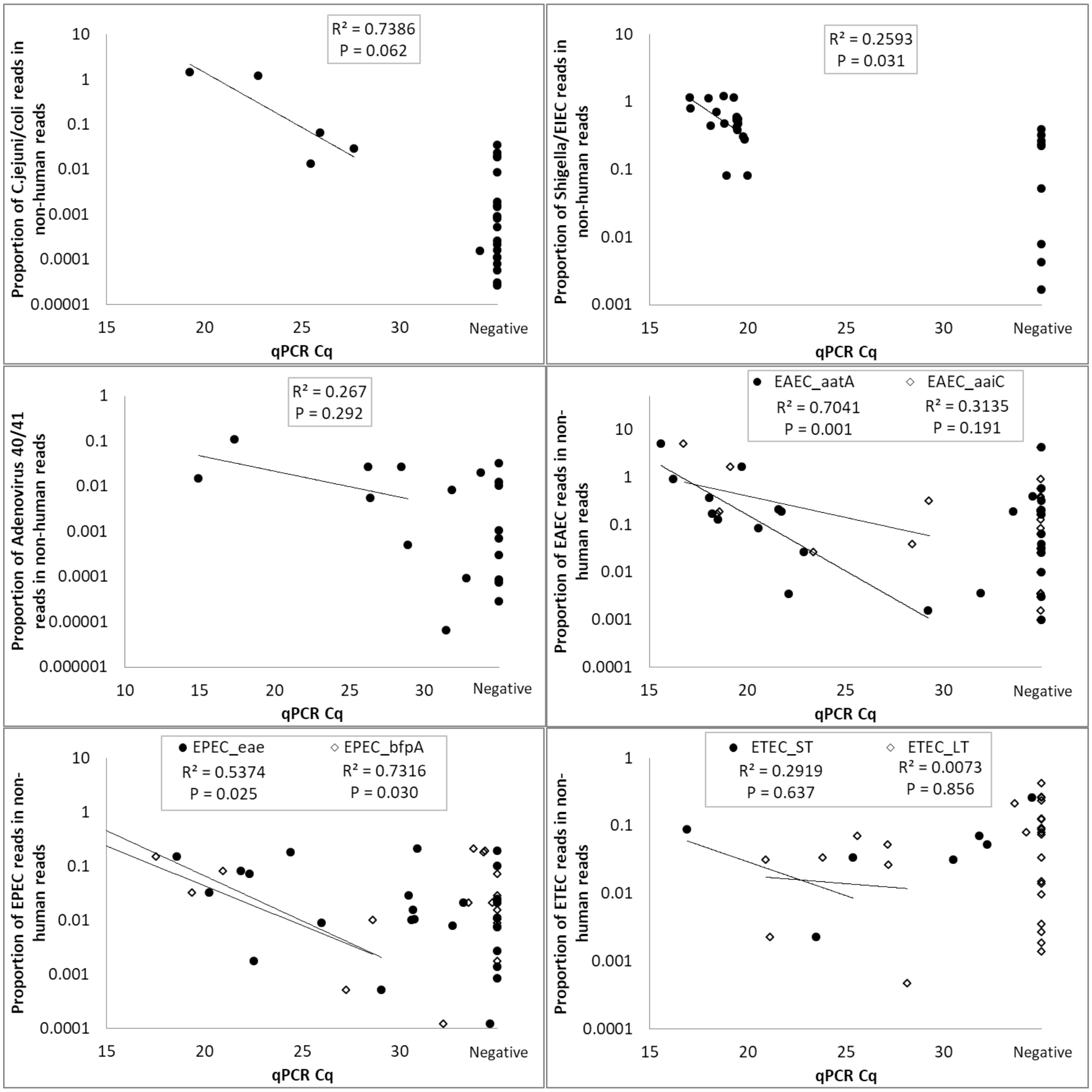

Reference

Hazen TH, Leonard SR, Lampel KA, Lacher DW, Maurelli AT, Rasko DA. 2016. Investigating the relatedness of Enteroinvasive Escherichia coli to other E. coli and Shigella Isolates by using comparative genomics. Infect Immun **84:**2362-2371.

Liu J, Platts-Mills JA, Juma J, Kabir F, Nkeze J, Okoi C, Operario DJ, Uddin J, Ahmed S, Alonso PL, Antonio M, Becker SM, Blackwelder WC, Breiman RF, Faruque AS, Fields B, Gratz J, Haque R, Hossain A, Hossain MJ, Jarju S, Qamar F, Iqbal NT, Kwambana B, Mandomando I, McMurry TL, Ochieng C, Ochieng JB, Ochieng M, Onyango C, Panchalingam S, Kalam A, Aziz F, Qureshi S, Ramamurthy T, Roberts JH, Saha D, Sow SO, Stroup SE, Sur D, Tamboura B, Taniuchi M, Tennant SM, Toema D, Wu Y, Zaidi A, Nataro JP, Kotloff KL, Levine MM, Houpt ER. 2016. Use of quantitative molecular diagnostic methods to identify causes of diarrhoea in children: a reanalysis of the GEMS case-control study. Lancet **388:**1291-1301.
